# Return of the Senior Editor of the “Journal” to Philadelphia

**Published:** 1901-05

**Authors:** 


### EDITORIALS.

### RETURN OF THE SENIOR EDITOR OF THE "JOURNAL" TO PHILADELPHIA.

We take pleasure in announcing the return of the senior editor of the Journal, Dr. Huidekoper, to Philadelphia after an absence of some ten years. In 1898 Dr. Huidekoper was Chief Surgeon of the First Army Corps and of the United States troops in Puerto Rico, and during the last two years has passed most of his time in Washington, D. C., devoting himself almost entirely to the interests of the legislation for an improved veterinary service in the army, so that the duties of conducting the Journal fell exclusively on the managing editor, Dr. Hoskins. We are glad to announce now that the combined energies of both the editors will from this time forward be devoted to the improvement of the Journal's pages.

Drs. Huidekoper and Hoskins have also combined in the practice of veterinary medicine, and while retaining the large hospital facilities of Dr. Hoskins' Philadelphia Veterinary Sanitarium at 3452 Ludlow Street, in West Philadelphia, they have opened a central office at 1304 Sansom Street to meet the convenience of their clients in the business centre of the city. This latter, which is but two blocks from the Pennsylvania and the Reading Railroad stations, will also be much more accessible to their professional friends who visit or pass through Philadelphia.

